# Upper-limb range of motion in children with cerebral palsy treated with botulinum neurotoxin: a population-based cohort study

**DOI:** 10.1186/s12891-026-09528-z

**Published:** 2026-01-26

**Authors:** Jenny Hedberg-Graff, Fredrik Granström, Marianne Arner, Elisabet Rodby-Bousquet, Lena Krumlinde-Sundholm

**Affiliations:** 1 Department of Women’s and Children’s Health, Karolinska Institutet, Stockholm, Sweden; 2 Department of Neurobiology, Health Sciences and Society, Karolinska Institutet, Stockholm, Sweden; 3 Center for Clinical Research, Region Värmland County Council, Karlstad, Sweden; 4 Department of Hand Surgery, Södersjukhuset, Stockholm, Sweden; 5 Department of Clinical Science and Education, Södersjukhuset, Karolinska Institutet, Stockholm, Sweden; 6 Centre for Clinical Research, Region Västmanland, Västerås, Sweden; 7 Department of Clinical Sciences, Orthopaedics, Lund University, Lund, Sweden

**Keywords:** Cerebral palsy, Children, Upper limb, Range of motion, Botulinum neurotoxin a

## Abstract

**Background:**

Our aim was to investigate change over time of passive range of motion (pROM) in the upper limbs of children with cerebral palsy (CP), treated or not treated with botulinum neurotoxin-A (BoNT-A).

**Methods:**

Data from 2000 to 2017 were collected from the Cerebral Palsy follow-up program and registry in Sweden (CPUP) for children with spastic or dyskinetic CP. Mixed models were used to analyse changes in pROM from the first, until the last measurement for five upper limb movements.

**Results:**

The study involved 496 children with CP, aged 1–15 years (median 2 years, Interquartile range = 4). Of these, 22% had received at least one BoNT-A treatment. Contractures were classified as red (severe) or yellow (moderate) based on the Traffic Light system within CPUP. About 36% developed upper limb contractures before age 15. Early BoNT-A treatment (< 4 years) implied better pROM outcomes over time compared with later treatment, after adjusting for pROM category, CP subtype and level of manual ability.

**Conclusions:**

Upper limb contractures can develop during growth in children with CP affecting one third of this population. Early monitoring of pROM can detect the first signs of muscle shortening before contractures are established. Our findings suggest that early BoNT-A treatment may help maintain pROM in children with CP.

**Supplementary Information:**

The online version contains supplementary material available at 10.1186/s12891-026-09528-z.

## Background

Cerebral palsy (CP) is the most common lifelong disorder of movement and posture affecting children and is classified according to its topographical involvement and type of movement disorder [1]. The majority of children with CP have a spastic (80%) or dyskinetic (∽10%) neurological subtypetype [2]. Spasticity and dyskinesia are described as consequences of the underlying brain injury [1] and are associated with contractures i.e., restricted passive range of motion (pROM) and impairment of motor function. The causes of movement restrictions are not fully understood, but are usually referred to as spasticity, torsional deformities, pain, muscle pathology and muscle weakness [3–5]. Muscle growth in children with CP may be affected already by the age of 15 months, with reduced volume, shorter length, fewer sarcomeres and reduced number of satellite cells [6]. Contracture development in the upper limb (UL) has been reported in one third of all children with CP and the pROM deterioration has been shown to start at an early age and continue to deteriorate during growth [7]. 

The first signs of muscle shortening are usually treated with orthoses and motor training activities [8, 9]. Over the past two decades, Botulinum neurotoxin A (BoNT-A) has been used as a treatment option for spastic and dyskinetic disturbances in children with CP. Treatment with BoNT-A into selected muscles, produces a dose-dependent chemical denervation resulting in reduced muscular activity in the target muscles [10]. Reduction of muscle overactivity in the spastic agonist [11] can facilitate orthotic treatment as well as enabling more effective active motion exercises of the antagonistic muscles groups [12]. Furthermore, BoNT-A treatment may delay the development of contractures and postpone potential surgery later in life [13]. Younger children could also be expected to achieve most clinical improvement in hand function development [14, 15]. 

The pharmacological effect of BoNT-A is reversible, lasting for approximately three months [14]. However, duration and effectiveness can be influenced by the child’s age, type of CP and level of manual ability [16]. Some studies have shown limited long-term effects of BoNT-A on pROM despite the reduction of muscle tone [17]. 

All children with CP in Sweden are invited to participate in the national Cerebral Palsy follow-up program and registry (CPUP) with an estimated coverage of 95% of children with CP born in the year 2000 and later [18]. In CPUP, standardized measurements of pROM are performed at regular intervals from diagnosis into adulthood [18]. In the present study, we wanted to examine if the development of pROM over time differed between children who had their first upper limb BoNT-A injection at an early age compared with children who were treated at a later age or those not treated with BoNT-A. Therefore, the aim of this study was to analyse changes in upper limb pROM over time in children with CP, who had been treated or not treated with BoNT-A, and if there were any differences if the first treatment with BoNT-A was given before 4 years of age, compared with treatment at 4–15 years of age.

## Methods

### Study design and ethics

This registry based cohort study includes CPUP [18] data from five of 21 regions in Sweden. Annual assessments from the CPUP upper limb protocol were carried out between 2000 and 2017 by occupational therapists at local paediatric habilitation units according to ordinary CPUP routines and schedule. Within CPUP, the assessment schedule is based on age and GMFCS levels. Children at GMFCS levels II to V are assessed more frequently than those at GMFCS level I. National guidelines for Botulinum Toxin A (BoNT-A) treatment have been established in Sweden and are based on specific clinical indications: (a) prevent contracture development and reduce pain (b) improvement of gross motor function, positioning, and reduction of dystonia/hyperkinesia and (c) promote hand function and fine motor skills. Data in this study was also used in a previous article reporting physical characteristics and factors related to upper limb treatment with BoNT-A in children with CP. [19] The study was approved by the Regional Ethical Review Board in Stockholm (Dnr 2013/1792-31/3).

### Participants

CPUP registry data for children with spastic or dyskinetic CP, aged 1–15 years, born between 2000 and 2017 and living in one of five regions in Sweden (Halland, Sörmland, Västmanland, Örebro and Västerbotten) were included in the study.

### Data and measurements

In this study, data were obtained according to CPUP upper limb protocol and comprised ages at different measurement occasions, age at first upper limb BoNT-A treatment in shoulder, elbow, forearm, wrist and finger flexor muscles, Manual Ability Classification System (MACS) level (i.e., ability to handle objects in daily life) [20] and pROM measurements. In addition, CP subtype (i.e., spastic unilateral CP, spastic bilateral CP or dyskinetic CP) were retrieved from the neuropediatric protocol [1, 21]. 

Passive range of motion (pROM) measures were retrieved from the first measurement occasion until the last measurement occasion for shoulder flexion/abduction (the mean values of shoulder flexion and shoulder abduction were used for further calculations), elbow extension, forearm supination and wrist extension with flexed and extended fingers. All measurements were made using a goniometer according to the Norkin and White guidelines [22]. These five selected movements are often described as affected by spasticity, causing the characteristic flexion-pronation posture and the affected muscles are also often chosen for BoNT-A treatment in the upper limbs. [5, 23]

The CPUP traffic light colour system was used to categorize pROM values. [7, 18, 19] The green category indicates full pROM, including a measurement error variance. In this study, a new, light-green category was added for pROM value within the green range but where a tightness at the end of the movement range was recognized [19]. The yellow category signifies moderate contracture, whereas red category indicates severe contracture. In this study, the red and yellow categories were considered as contractures i.e., restricted pROM. Ranges for the different upper-limb joint motions according to the traffic-light system are presented in Supplementary Table 1.

### Statistical analysis

Distributions of the variables were examined, and categorical data and ordinal data were presented as frequencies (n) and percentages (%). Continuous data were presented as medians and interquartile range (IQR) due to the non-symmetrical distribution of the variables.

Mixed models were used to analyse pROM changes over time (expressed as degrees of the movement range) for five upper limb joints and to compare pROM changes over time in children who had received BoNT-A treatment with children who had not. Analysis was conducted in 3-year intervals (1–3, 4–6, 7–9, 10–12 and 13–15 years of age) and compared pROM changes over time in three different groups: (i) children with first time UL BoNT-A treatment early (at 1–3 years of age), (ii) children with first time UL BoNT-A treatment later (at 4–15 years of age) and (iii) children not treated with UL BoNT-A at any time (at 1–15 years of age). In children who received BoNT-A, spastic muscles that impeded movements such as shoulder flexion/abduction, elbow extension, forearm supination, wrist extension with flexed and wrist extension with extended fingers were treated with UL BoNT-A after clinical requirements. Pairwise comparison of pROM development over time between the three groups were made with a significance level set at *P*-value of less than or equal to 0.05. Group was used as a fixed variable and child as random variable. A heterogeneous autoregressive variance structure of the random effects has been assumed in the model specifications because the same child can have several measurement occasions in the same age range [24]. The analysis was adjusted for MACS level, CP subtype, and pROM category at first measurement occasion.

In all analyses, only one side, right or left, per child was used. For children where only one side was treated with UL BoNT-A, that side was analysed. In cases where both sides were treated, the side with the lowest range of motion value was used. IBM SPSS Statistics for Windows, Version 22.0. Armonk, NY: IBM Corp. was used in all analyses in this study.

## Results

In total, 3756 measurements were reported for 496 children with spastic or dyskinetic CP (317 males, 179 females) of which 63% (*n* = 311) had their first measurement occasion at the age of 1–3 years. The children’s age at first measurement i.e., when entering the CPUP was median 2 years, IQR = 4, range = 1–14 years. Among children not BoNT-A treated (*n* = 388), *n* = 232 (60%) had their first measurement occasion at 1–3 years of age, while among children BoNT-A treated (*n* = 108), *n* = 79 (73%) had their first measurement occasion at 1–3 years of age. Within the entire population, 22% (*n* = 108) had at least one occasion with BoNT-A treatment over time, with a total of 324 UL BoNT-A occasions (1–3 y, *n* = 172, mean = 3.5, range 1–5; 4–15 y *n* = 152, mean = 2.6, range 1–5). Age at the first BoNT-A treatment of the upper limb was median 4 years, IQR = 4.7, range = 1–15 years. Almost half, (45%, *n* = 49) already had their first BoNT-A treatment at 1–3 years of age. A similar proportion of boys (*n* = 71, 22%) and girls (*n* = 37, 21%) were treated with UL BoNT-A (Table 1).

**Table 1:** Distribution of sex, CP-subtype, MACS level, passive range of motion category at the *first* and *last* measurement in three groups, children first treated at 1–3 years of age, children first treated at 4–15 years of age and children not treated with UL BoNT-A

Variables	UL BoNT-A		No UL BoNT-A	Total
	BoNT-A 1–3-year *n* (%)	BoNT-A 4–15-year *n* (%)	No BoNT-A 1–15-year *n* (%)	Total *n* (%)
Girls	19 (39)	18 (31)	142 (37)	179 (36)
Boys	30 (51)	41 (69)	246 (63)	317 (64)
Total CP-subtype	49 (10)	59 (12)	388 (78)	496 (100)
Bilateral Spastic CP	19 (39)	29 (49)	215 (55)	263 (53)
Unilateral Spastic CP	27(55)	23 (39)	134 (35)	184 (37)
Dyskinetic CP	3 (6)	7 (12)	39 (10)	49 (10)
Total MACS- levels	49 (10)	59 (12)	385 (78)	493 (100)
MACS I	6 (12)	0 (0)	142 (37)	148 (30)
MACS II	12 (24)	17 (29)	89 (23)	118 (24)
MACS III	15 (31)	12 (20)	55 (14)	82 (17)
MACS IV	6 (12)	11 (19)	38 (10)	55 (11)
MACS V	10 (20)	19 (32)	61(16)	90 (18)
Total GMFCS-levels	48 (10)	59 (12)	381 (78)	488 (100)
GMFCS I	23 (48)	19 (32)	172 (45)	214 (44)
GMFCS II	4 (8)	6 (10)	59 (15)	69 (14)
GMFCS III	4 (8)	1 (2)	43 (11)	48 (10)
GMFCS IV	11(23)	11(19)	50 (13)	72 (15)
GMFCS V	6 (13)	22 (37)	57 (15)	85 (17)
Total passive range of motion (pROM)	49 (10)	59 (12)	388 (78)	496 (100)
Passive ROM green, ^*a*^ *first*	15 (31)	7 (12)	209 (54)	231 (47)
Passive ROM light green, *first*	23 (47)	22 (37)	105 (28)	150 (30)
Passive ROM yellow, *first*	9 (18)	18 (31)	56 (14)	83 (17)
Passive ROM red, *first*	2 (4)	12 (20)	18 (5)	32 (6)
Passive ROM green, ^*b*^ *last*	14 (29)	7 (12)	208 (54)	229 (46)
Passive ROM light green, *last*	11 (22)	7 (12)	72 (19)	90 (18)
Passive ROM yellow, *last*	21 (43)	19 (32)	85 (22)	125 (25)
Passive ROM red, *last*	3 (6)	26 (44)	23 (6)	52 (10)
BoNT-A occasions n (%)	172 (53)	152 (47)	0	324 (100)
Mean BoNT-A occasions	3.5	2.6	0	0.7
Measurement occasions	637 (17)	763 (20)	2356 (63)	3756 (100)

^*a*^*First-* first measurement at entering CPUP, ^*b*^*Last-* Last measurement

*MACS *Manual Ability Classification System*; GMFCS *Gross Motor Function Classification System,* UL BoNT-A *upper limb Botulinum neurotoxin-A

Distribution within CP-subtypes, MACS levels and pROM categories are presented for children who were treated for the first time with UL BoNT-A at 1–3 years of age (median = 2 years, IQR = 1 year, range = 1–3 years), at 4–15 years of age (median = 7 years, IQR = 6 years, range = 4–15 years) and for children not treated with UL BoNT-A (Table 1).

In total, most children had full pROM within green category values both at their first (77%) and at the latest (64%) pROM measurement occasion. The highest proportion of contractures were demonstrated in children treated with UL BoNT-A at 4–15 years of age, both at their first and latest measurement occasion in all movements, while the lowest proportion of contractures in all joints and movements were demonstrated in children not treated at both first and last measurement (Table 1, Supplementary Fig. 1).

The median time between entering CPUP and the first UL BoNT-A treatment was shorter for children first treated at 1–3 years (median time 15 months) compared with children treated for the first time at 4–15 years (median time 35 months). Children who received their first UL BoNT-A at 1–3 years of age also entered the CPUP registry at earlier age (median = 1 years, IQR = 0 years, range = 0–3 years) compared with children who received their first BoNT-A treatment later, at 4–15 years of age (median = 3 years, IQR = 5 years, range = 0–14 years).

**Fig. 1:**
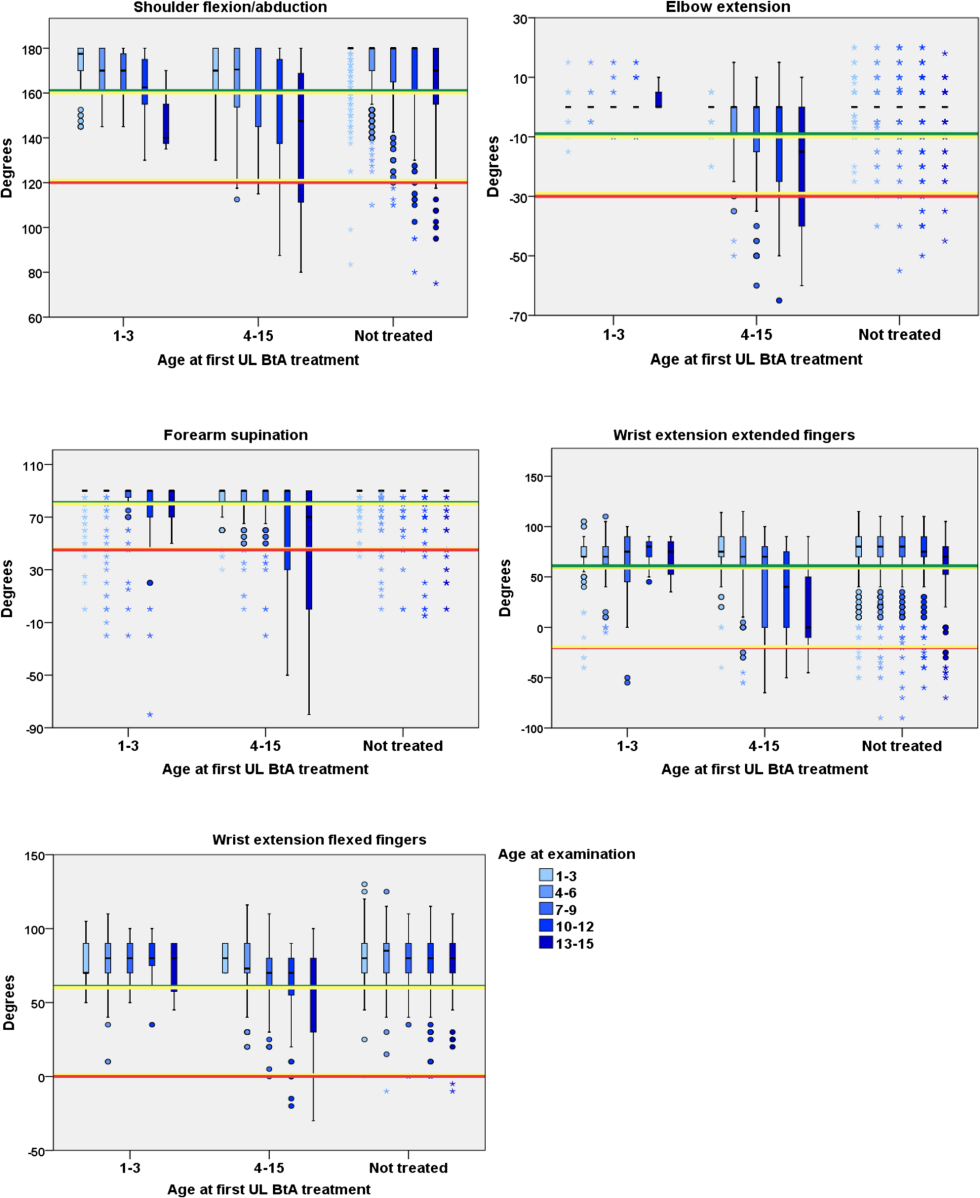
Passive range of motion for children treated or not treated with upper limb BoNT-A Passive range of motion in degrees for upper-limb movements at the latest measurement distributed across children first treated with UL BoNT-A at 1–3 years, at 4–15 years and children not treated. The median is marked inside the box. The length of whiskers is determined by multiplying the interquartile range by a factor of 1.5. Observations/ measurement outside of the whiskers are outliers and marked with a circle (o). Outliers outside a range of 3 times the interquartile range are depicted with an Asterix (*). The colored horizontal lines in the figures indicate the critical values according to CPUP traffic light system. The green line indicates full pROM, yellow line indicates a moderate contracture, and red line indicates severe contracture. In this study, values within the red and yellow lines were considered as contractures i.e., restricted pROM

In all investigated movements, the mean pROM deteriorated with increasing age and the decline started already at 4–6 years of age for shoulder flexion/abduction (*P* < 0.01) and for wrist extension with extended fingers (*P* < 0.01), at 7–9 years for forearm supination (*P* < 0.01) and elbow extension (*P* = 0.01), and at 13–15 years for wrist extension with flexed fingers (*P* = 0.03). The deterioration over time was most pronounced for wrist extension with extended fingers (*P* < 0.01) (Fig. 1; Table 2).

**Table 2:** Differences in passive range of motion (in degrees) across age groups in five upper limb movements of 496 children

				95% Confidence Interval for Difference	
Age years	Reference	Mean Diff	*P*-value	Lower Bound	Upper Bound
^a^ Shoulder					
4–6	1–3	-2.5^*^	< 0.01	-4.5	− 0.5
7–9	1–3	-5.5^*^	< 0.01	-8.3	-2.7
10–12	1–3	-12.5^*^	< 0.01	-16.5	-8.5
13–15	1–3	-19.0^*^	< 0.01	-25.4	-12.6
^a^ Elbow					
4–6	1–3	0.0	0.99	− 0.8	0.8
7–9	1–3	-1.2	0.11	-2.6	0.3
10–12	1–3	-1.7	0.11	-3.8	0.4
13–15	1–3	-4.4^*^	0.01	-7.8	-1.0
^a^ Forearm supination					
4–6	1–3	− 0.7	0.27	-1.9	0.5
7–9	1–3	-2.5^*^	0.01	-4.3	− 0.8
10–12	1–3	-8.0^*^	< 0.01	-11.1	-5.0
13–15	1–3	-13.5^*^	< 0.01	-19.7	-7.3
^a^ Wrist extension flexed fingers					
4–6	1–3	− 0.3	0.74	-2.0	1.4
7–9	1–3	-1.1	0.37	-3.4	1.3
10–12	1–3	-4.1^*^	0.03	-7.8	-0.3
13–15	1–3	-7.5^*^	0.03	-14.2	-0.9
^a^ Wrist extension extended fingers					
4–6	1–3	-5.3^*^	< 0.01	-8.5	-2.0
7–9	1–3	-12.8^*^	< 0.01	-17.6	-8.0
10–12	1–3	-15.3^*^	< 0.01	-22.1	-8.5
13–15	1–3	-24.2^*^	< 0.01	-36.0	-12.4

Based on estimated marginal means*. The mean difference is significant at the 0.05 level. ^a^ Dependent variable: degree

Children who were not treated (*n* = 388) with UL BoNT-A generally maintained their pROM within the green range (full pROM) for most movements (four of five) over time. These children generally have favourable movement development over time in forearm supination compared to children BoNT-A treated. However, in children not treated contractures developed in wrist extension with extended fingers at older ages. Children with a first time UL BoNT-A treatment at 1–3 years of age (*n =* 49) maintained their mean pROM over time within the green range (full pROM) in three (elbow extension, wrist extension with flexed fingers and wrist extension with extended fingers) out of five movements. Children with a first treatment at 4–15 years of age (*n* = 59) showed a significant decline of pROM over time in all movements (Fig. 1).

Moreover, the decline in pROM over time differed among children who were not treated, those who received their first UL BoNT-A treatment early (1–3 years) and those treated later (4–15 years). Children who had their first treatment at older age showed a more significant pROM deterioration in four movements: shoulder flexion (*P* < 0.01), forearm supination (*P* < 0.01), wrist extension with flexed fingers (*P* = 0.01), and wrist extension with extended fingers (*P* = 0.04), compared with those who were not treated with UL BoNT-A. Additionally, the group treated at older age showed a more pronounced decline in pROM for wrist extension with flexed fingers (*P* = 0.02) and in wrist extension with extended fingers (*P* = 0.08) compared with those who received their first BoNT-A treatment early (1–3 years) (Table 3).

**Table 3:** Mixed model analysis of changes in passive range of motion (mean degrees, range, *P*-value) in children treated with botulinum neurotoxin-A in the upper limb (UL BoNT-A) at 1–3 years, 4–15 years or not treated

Movement/pROM	Shoulder flexion/abd	Elbow extension	Forearm supination	Wrist/flex fingers	Wrist/ext fingers
Groups Mean diff degrees (95% CI) (*P*-value)					
No UL BoNT-A treatment	Ref	Ref	Ref	Ref	Ref
UL BoNT-A at 1–3 y of age	-3.1 (-9.7, 3.6) (P = 0.36)	1.1 (-2.9,5.1) (P = 0.58)	-6.8 (-12.0, -1.7) (P = 0.01)	-0.0 (-6.0, 6.0) (P = 0.9)	3.7 (-6.5, 13.9) (P = 0.47)
UL BoNT-A at 4–15 y of age	-8.6 (-12.7, -4.5) (P < 0.01)	-1.8 (-4.3, 0.6) (P = 0.14)	-10.8 (-14.8, -6.8) (P < 0.01)	-7.8 (-11.4, -4.2) (P < 0.01)	-6.5 (-12.9, -0.1) (P = 0.04)
UL BoNT-A at 1–3 y of age	Ref	Ref	Ref	Ref	Ref
UL BoNT-A at 4–15 y of age	-5.5 (-13.1, 2.1) (P = 0.15)	-2.9 (-7.5, 1.6) (P = 0.20)	-4.0 (-10.2, 2.2) (P = 0.20)	-7.8 (-14.4, 1.2) (P = 0.02)	-10.2 (-21.5, 1.0) (P = 0.08)
No UL BoNT-A treatment	3.1 (-3.6, 9.7) (P = 0.36)	-1.1 (-5.1,2.9) (P = 0.58)	6.8 (1.7, 12.9) (P = 0.01)	0.0 (-6.0, 6.0) (P = 0.9)	-3.7 (-13.9, 6.5) (P = 0.47)

The analysis is adjusted for level of the Manual Ability Classification System, CP subtype and passive range of motion category at the first measurement

## Discussion

This study describes a population-based cohort of 496 children with CP (1–15 years of age) regarding their change of pROM over time, for those receiving and those not receiving UL BoNT-A treatment. Additionally, we investigated possible differences in pROM development in children who had not received UL BoNT-A treatment at all, and children who had received their first UL BoNT-A at an early age or at a later age. In this population of children with CP, the majority (78%) did not receive UL BoNT-A treatment. Children who were not treated with BoNT-A kept most of their pROM values within the green (normal) movement range over time, while children treated with BoNT-A developed contractures. This is not surprising as children with resistance during passive range of motion are at higher risk of contracture development compared to children without. However, children treated with UL BoNT-A before four years of age, had favourable pROM development over time compared with those treated for the first time at a later age, also when adjusting for CP subtype, MACS level and for pROM at their first measurement occasion.

Using the “traffic-light” categorisation system in the CPUP registry, 36% of the children showed signs of contractures (yellow or red values) in the ULs. This is consistent with previously reported results in a partly different geographic cohort in Sweden [7]. Studies have shown that pROM starts to decrease at an early age both in the upper and lower limbs [25, 26]. Recent research of the lower limb also showed that 44% of the children who developed a first contracture also developed secondary contractures in other movements [27]. Together, these findings suggest that affected pROM should be treated early [26]. 

Interestingly, 78% of children receiving BoNT-A treatment for the first time at an early age demonstrated full ROM during their initial measurement. Among these children, only 6% developed severe contractures later in life. In contrast, barely half (49%) of the children treated after the age of four showed full ROM initially, and 44% of them developed severe contractures later in life. These results support previous assumptions that once a contracture is established it is difficult to stop and that children with CP seem to have the best benefits of BoNT-A at a young age to prevent contractures [15, 17]. Even so, it is important to recognize that children who receive their first BoNT-A treatment after developing a contracture may still benefit from BoNT-A treatment. This treatment option may assist in maintaining their existing pROM, reducing pain and facilitating care. However, recent findings indicate that the use of BoNT-A for spasticity management in children with CP may increase muscle atrophy and fibrofatty content in the muscle [28]. Considering the reduced muscle growth in CP with smaller, shorter and weaker muscles, the benefits and consequences of spasticity management has to be considered in all children [28]. According to our findings, early BoNT-A treatment seems to be more beneficial for development of pROM over time. Consequently, ongoing monitoring of pROM remains crucial for all children with CP.

The reason why some children, but not all, develop severe contractures is a debated topic. We know that muscles in children with CP are different compared with muscles in typically developed children, [29] but we lack knowledge about how BoNT-A affects muscles in children with CP over a longer period of time. New evidence suggests that children with CP have a muscle growth delay which likely contributes to the development of contractures [30]. Initially, muscle growth in children with CP follows that of typically developed children, but the growth seems to decrease as early as at 15 months [31]. However, interventions including active movements that stimulate muscle growth in young children with CP may be an important contributor in preventing contractures [31]. To keep contractures from developing, it can be beneficial to pay attention to ROM variations even when the children only have mild signs of muscle shortening, for example, in the case of full pROM with tightness at the end of the movement range. Subsequently, to promote pROM development, active voluntary movements in daily life also need to be encouraged at an early age to promote muscle growth and also maintain muscle length [31, 32]. Systematic registration of active ROM may be a complementary aid before decisions on further intervention strategies are made.

Given that the majority of children did not develop contractures over time, we can assume that therapists and hand surgeons were good at identifying and selecting those children who would benefit most from BoNT-A treatment in the upper limbs in this population. The findings of the present study indicate that initiation of the UL BoNT-A treatment in children identified as appropriate candidates at an early age, before contractures have developed, is crucial. In this regard, the new light-green category in the CPUP traffic light system is of particular interest since this category indicates a first sign that a muscle is at risk of a contracture. Thus, the light-green category may be an important indicator for guiding further treatment. Subsequently, more research of muscles in children with CP are warranted.

In this study, a significant pROM deterioration was found in four out of five investigated joint movements in children undergoing first time UL BoNT-A treatment at 4–15 years of age compared with children who had never been treated. Among the investigated movements, the most affected movements were observed in the shoulder and the wrist regardless of whether they had been BoNT-A treated or not which also was confirmed in a recent study showing that shoulder contractures were found to increase continuously with age across all MACS levels [33]. However, this study shows that wrist extension with simultaneously extended fingers had the most pronounced pROM deterioration over time in all children, regardless of whether they had been BoNT-A treated or not. It has been reported that flexor digitorum profundus and superficialis muscles are often the first to be affected by restricted pROM, already occurring below four years of age [7]. However, only 20% of the BoNT-A treatments targeted the finger flexors [19]. Given this knowledge, finger flexors probably need more attention early in these children’s lives.

Other important muscles for hand function are the thumb muscles; it has been reported that 40% of all children with CP show signs of a spastic thumb-in-palm deformity [34]. Valid and reliable methods to detect deformities of the thumb are lacking, according to a Cochrane report [35]. The House classification describing different patterns of muscle involvement in thumb-in-palm has been shown to have low inter-rater reliability and it is neither designed nor useful for following pROM development over time [36]. Goniometric measurements of thumb motion are difficult to perform in children with limited pROM and have not been regarded as possible to include in a broad follow-up program as the CPUP. We are currently underway to study the reliability of a new and easier method for evaluating and following radial abduction in the thumb [37]. Since the muscles of the thumb are among the most often treated at the first BoNT-A occasion and since they have a central role in grip function, it would have been very interesting to study their development over time.

This study has several limitations. The mixed model analysis was used to analyse pROM changes over time (expressed as degrees of the movement range) adjusted for the variables CP subtype, MACS level and pROM category i.e., ROM at first measurement occasion. Moreover, most children treated with BoNT-A in Sweden are also routinely followed up and treated according to national guidelines with conservative treatment, for example, through active use of the upper limbs in daily activity. However, the analysis is not adjusted for other interventions the child might have had in addition to UL BoNT-A treatment. Therefore, we do not know whether other interventions may have affected the results to some extent. Unfortunately, this study did not control for surgical or orthotic treatment, nor did it include assessments of spasticity or measures of active movement since these data were poorly or not reported in the CPUP registry and not available for this study.

As we can assume that the children who had contractures at their first measurement were also the ones who developed even more severe contractures over time, we adjusted the analyses for level of pROM at the first measurement. However, the age at the first measurement varied somewhat, as some of the children treated for the first time at later age (4–15 years of age) entered CPUP when they were older than the usual age of entering. It seems likely that children who have moved to Sweden from other countries often are enrolled in the registry once they come to Sweden, which typically means they are included in the CPUP program at a later age. Still, among all children (*n* = 496), *n* = 311 (63%) had their first measurement i.e., entered CPUP at the age of 1–3 years. Nevertheless, the adjustment for level of pROM at the first measurement biases to some extent the age effect of the pROM development in the analyses. However, we do not expect the related bias to significantly influence the findings.

## Conclusions

Upper limb contractures can develop over time as children with CP grow, affecting one-third of the children in this population-based study. By systematic monitoring of pROM from an early age, early signs of muscle shortening such as tightness at the end of pROM, can be detected before contractures develop. Our findings demonstrate that pROM in children who received BoNT-A treatment after the age of four years had a more pronounced decline in pROM over time compared to those treated at an earlier age or not treated in the upper limbs. These findings underscore the importance of careful patient selection and a clearly defined therapeutic objective to optimize the benefits of BoNT-A treatment in children with cerebral palsy. Subsequently, early intervention with BoNT-A may be beneficial to maintain upper limb pROM in children with CP.

## Supplementary Information

Supplementary Material 1.

## Data Availability

The data that support the findings of this study are available from the CPUP registry, but restrictions apply to the availability of these data, and they are not publicly available. Data are however available from the authors upon reasonable request and with permission of KVB Region Skåne [https://www.skane.se/om-region-skane/forskning/for-dig-som-forskar/personuppgifter-och-patientdata/kvb-ansokan-for-utlamnande-av-patientdata/].

## Abbreviations

BoNT-A: Botulinum Neurotoxin-A
CP: Cerebral Palsy
CPUP: Cerebral Palsy Follow-up Program
GMFCS: Gross Motor Function Classification System
IQR: Interquartile Range
MACS: Manual Ability Classification System
pROM: Passive Range of Motion
UL: Upper limb
