# Supplementary material for: Upper-limb range of motion in children with cerebral palsy treated with botulinum neurotoxin: a population-based cohort study
Source: BMC Musculoskelet Disord. 2026 Jan 26;27:65. doi: 10.1186/s12891-026-09528-z (PMC12849247; doi:10.1186/s12891-026-09528-z)

SUPPLEMENTARY TABLES AND FIGURES

Supplementary Table 1. Critical values CPUP traffic light system.

Movement	Red/ contracture	Yellow/ contracture	Green/ tightness	Green/ normal
Shoulder flexion	≤ 120°	> 120° < 160°	≥ 160°	≥ 160°
Elbow extension	≤ -30°	> -30° < -10°	≥ -10°	≥ -10°
Forearm Supination	≤ 45°	> 45° < 80°	≥ 80°	≥ 80°
Wrist extension/ flexed fingers	< 0°	> 0° < 60°	≥ 60°	≥ 60°
Wrist extension/ extended fingers	≤ -20°	> -20° < 60°	≥ 60°	≥ 60°

Supplementary Figure 1. Traffic light system with pROM for children treated or not treated with BoNT-A.

Proportion of passive range of motion (pROM) categories according to the traffic light system (red, yellow, green), presented for three groups of children based on the time of first treatment with Botulinum neurotoxin A in the upper limb (UL BoNT-A). Passive ROM at their first and last measurement shown for children first treated with UL BoNT-A at 1-3 years of age, children first treated at 4-15 years of age and children not treated with UL BoNT-A.

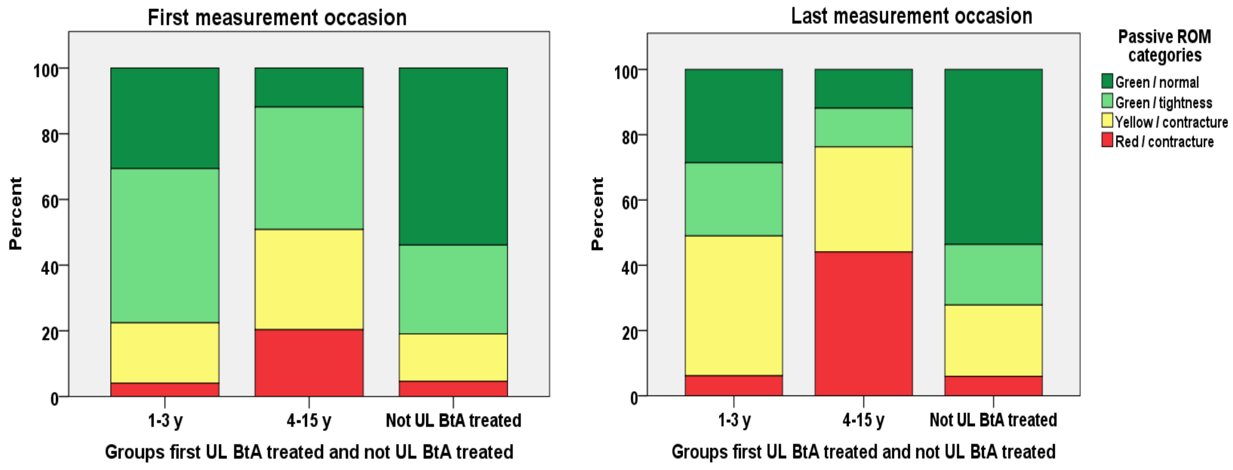

Supplement: Supplementary file 1 — Supplementary Material 1. [file 12891_2026_9528_MOESM1_ESM.pdf]
